# Evaluation of Safety of Disposable Saliva Ejectors after Autoclaving Sterilisation

**DOI:** 10.3290/j.ohpd.c_2088

**Published:** 2025-07-04

**Authors:** Hye-Young Yoon, / Sun-Jung Shin, / Bo-Mi Shin, / Hyo-Jin Lee, / Jin-Sun Choi, / Soo-Myoung Bae

**Affiliations:** a Hye-Young Yoon Assistant Professor, Department of Dental Hygiene, College of Dentistry, Gangneung-Wonju National University; Research Institute of Oral Science, Gangneung-Wonju National University; Research Institute of Dental Hygiene Science, Gangneung-Wonju National University, Gangneung, Korea. Conceptualisation, data acquisition, formal analysis, wrote original draft, reviewed and edited manuscript.; b Sun-Jung Shin Professor, Department of Dental Hygiene, College of Dentistry, Gangneung-Wonju National University; Research Institute of Oral Science, Gangneung-Wonju National University; Research Institute of Dental Hygiene Science, Gangneung-Wonju National University, Gangneung, Korea. data acquisition, formal analysis, reviewed and edited manuscript.; c Bo-Mi Shin Professor, Department of Dental Hygiene, College of Dentistry, Gangneung-Wonju National University; Research Institute of Oral Science, Gangneung-Wonju National University; Research Institute of Dental Hygiene Science, Gangneung-Wonju National University, Gangneung, Korea. data acquisition, formal analysis, reviewed and edited manuscript.; d Hyo-Jin Lee Associate Professor, Department of Dental Hygiene, College of Dentistry, Gangneung-Wonju National University; Research Institute of Oral Science, Gangneung-Wonju National University; Research Institute of Dental Hygiene Science, Gangneung-Wonju National University, Gangneung, Korea. data acquisition, formal analysis, reviewed and edited manuscript.; e Jin-Sun Choi Assistant Professor, Department of Dental Hygiene, College of Dentistry, Gangneung-Wonju National University; Research Institute of Oral Science, Gangneung-Wonju National University; Research Institute of Dental Hygiene Science, Gangneung-Wonju National University, Gangneung, Korea. data acquisition, formal analysis, reviewed and edited manuscript.; f Soo-Myoung Bae Professor, Department of Dental Hygiene, College of Dentistry, Gangneung-Wonju National University; Research Institute of Oral Science, Gangneung-Wonju National University; Research Institute of Dental Hygiene Science, Gangneung-Wonju National University, Gangneung, Korea. Conceptualisation, data acquisition, formal analysis, supervision, wrote original draft, reviewed and edited manuscript.

**Keywords:** contamination, dental infection control, proteins, reuse, saliva ejector.

## Abstract

**Purpose:**

To identify the bacteria and proteins that remain after cleaning and sterilisation of SEs used in dental practices, and to investigate potential problems when reusing SEs.

**Materials and Methods:**

In total, 105 SEs used on study participants were collected. The collected SEs were immediately immersed in the disinfectant solution and then washed with tap water and a cleaning brush. The SEs were dried, placed in sterile pouches, and sterilised in an autoclave before being used in the experiment. To detect residual bacteria, SE samples were cultured in brain heart infusion (BHI) broth for 10 days, followed by re-culturing on blood agar and BHI agar. Bacterial identification was performed using bacterial colonies. To identify residual proteins, SE samples were stained with phloxine B, and the stained sites and area were analysed.

**Results:**

Residual bacteria were found in one (1.64%) of 61 sterilised SEs. The cultured colonies were identified as *Staphylococcus warneri*. Residual proteins were observed on the tips of 36 (81.8%) of the 44 SE samples, and on the bodies of all samples (100%). The average stained area of the residual proteins on the SE bodies was 1.78% (standard deviation, 3.1%).

**Conclusion:**

The presence of bacteria and proteins in sterilised SEs indicates that their reuse can cause cross-contamination. This study is the first attempt to provide experimental evidence of the problems with reuse of SEs.

With the emergence and spread of COVID-19, awareness of and interest in infection control within medical facilities, including dental offices, have increased along with the demand for safer medical practices.^8,26^ With these societal changes, dental professionals need more information to recognise the importance of infection control practices, as well as how to implement and evaluate them correctly. Controlling infection using dental instruments and materials is important. Previous studies have shown that people are more careful about the disinfection of dental instruments after receiving information about cross-infections in the dental office, and many people indicated that they avoided visiting the dental office because they were worried about becoming infected from the dental chair or instruments during COVID-19.^14,19^ Because most instruments and materials used in dental offices come into contact with patients’ mucous membranes and teeth, and are frequently contaminated with saliva and blood, improperly treated dental instruments and materials can act as vectors for the transmission of pathogenic microorganisms.^17,23^

Dental instruments are categorised as high-risk, moderate-risk, and non-risk based on the purpose of use and risk of infection. It is recommended to sterilse high-risk and moderate-risk instruments if they are heat-resistant; however, if they are not heat-resistant and cannot be sterilised, they are recommended to be used once and then discarded.^21^ Disposable dental devices include syringe needles, prophylaxis cups, prophylaxis brushes, saliva ejectors, high-volume evacuator tips, etc.^17,23^

Plastic saliva ejectors (SE) are frequently used in dental procedures to prevent the airborne spread of aerosols and droplets containing the patient’s saliva or blood during procedures involving ultrasonic scalers and high-speed handpieces.^1^ SEs can come into contact with a patient’s mucous membranes and teeth, including potentially damaged mucous membranes, making them high- or moderate-risk instruments.^23^ According to recommended infection control practices for high- or moderate-risk instruments, SEs should be sterilised if possible; however, because they are made of plastic and are not heat-resistant, and because their narrow inner diameter of approximately 6 mm makes it difficult to clean them thoroughly, they are recommended to be used once and discarded.^17,21,23^ However, previous studies examining dental instrument reprocessing practices showed that SEs are reused after sterilisation, and recent studies in Korea have also found that SEs are reused after sterilisation, though at a lower rate.^6,13,15,23^

Previous studies^2,27^ that identified risks from reprocessing healing abutments (HA), a material used to aid in recovery after dental implant surgery, found that HA are a high-risk device which are difficult to completely clean and disinfect. Therefore, although they should be discarded after single use,they are being sterilised and reused. The same studies^2,27^ also found that bacteria and proteins remained in used HA even after sterilisation, indicating that cross-infection with bacteria or proteins can occur. In particular, the residual protein suggests that cross-infection with prions – protein-based infectious particles which are highly resistant to proteolytic enzymes and heat and can cause neurodegenerative diseases including spongiform encephalopathy (Creutzfeldt-Jakob disease) – is possible.^18,25^

Similar to the findings regarding the risks of sterilised and reused HA, it is possible that residual bacteria or proteins may be found in sterilised and reused SE, indicating the potential for cross-infection from sterilised SE.

Therefore, this study aims to determine the presence of residual bacteria and proteins after sterilisation of SEs and to provide an experimental basis determining the risk of reusing SEs after sterilisation.

## MATERIALS AND METHODS

### Study Participants

This study was conducted at the Department of Dental Hygiene, Gangneung-Wonju National University, Korea, after obtaining approval from the Public Institute Review Board (P01-202303-06-001). In this study, the safety of SEs after sterilisation was evaluated to determine the presence of microorganisms in the sterilised SEs. In one previous study,^25^ 55 samples were collected from sterilised HAs to determine residual microorganisms, and in another,^18^ 40 samples were collected from sterilised HAs to determine residual organisms. Based on these studies, 105 samples (residual microbial assessment: 61 samples; residual organic assessment: 44 samples) were collected, taking a 10% dropout rate into account.

The participants were recruited, scaling was performed, and SEs were collected. Adults aged ≥19 years who voluntarily visited the study center and agreed to participate were included in the study, with the exception of those with tooth hypersensitivity, complained of hypersensitivity during scaling, and students in the researchers’ department.

### Sample Collection and Treatment

Samples were collected from March 16, 2023 to January 18, 2024. Scaling was performed in the participants for approximately 30 min, and the SEs used were included in the study. A total of 105 SEs (Asa Dental; Massarosa, Italy) were collected from the participants.

The collected SEs were immediately immersed in a disinfectant solution (2% quaternary ammonium compounds; IL-CHUNG DENTAL, Seoul, Korea) for 2 h as recommended by the manufacturer, and then washed with tap water and a cleaning brush. After washing and drying at room temperature, SEs were placed in sterile pouches and sterilised in an autoclave (HTA-50V; Han Sung, Cheonan, Korea). Sterilised SEs were packed in sterile pouches and stored at room temperature until use.

### Evaluation of Residual Bacteria on Sterilised SE

#### Confirmation of bacterial growth

Of the 105 sterilised SEs, 61 were removed from the sterile pouch using sterile tweezers under aseptic conditions and placed in a sterile test tube containing 10 ml of brain heart infusion broth (BHI; MB cell, Seoul, Korea). A new and unused SE straight out of the package was used as a negative control to confirm residual bacteria. The tubes were incubated in a 37°C and 5% CO_2_ in an incubator (MCO-170AIC, Panasonic, Osaka, Japan) for 10 days and examined for changes in turbidity.^2^ After 10 days of incubation, 100 μl of the liquid culture was inoculated onto BHI agar (BHIA, MB cells) and blood agar (BA, Hangang, Gunpo, Korea), and then incubated for 48 h in a 37°C, 5% CO_2_ incubator (Panasonic) for BHIA and a 37°C incubator (H2200-HE, Benchmark Scientific; Sayreville, NJ, USA) for BA.

#### Identification of bacterial species

Only one sample formed colonies on solid media and these colonies were subjected to bacterial identification. Four colonies per morphotype were selected from the BHIA-grown colonies and two colonies from the BA-grown colonies for pure culture. Then, the pure-cultured bacterial colonies were suspended in 200 μl sterile distilled water. Genomic DNA was extracted from this suspension using a G-spin Genomic DNA Extraction Kit (Intron Biotechnology; Seongnam, Korea) according to the manufacturer’s instructions. The 16S rRNA gene was amplified in QuantStudio 7 flex (Applied Biosystems; Bedford, MA, USA) using PCR primers (27F; 5’-AGA GTT TGA TCM TGG CTC AG-3,’ 1492R; 5’-TAC GGY TAC CTT GTT ACG ACT T-3’) and Solg 2x Taq PCR Pre-Mix (SolGent; Daejeon, Korea). The PCR conditions were as follows: 0.1 μM forward and reverse primers and 100 pg of bacterial genomic DNA were added, distilled water was added to a final volume of 30 μl, and the reaction was performed at 94°C for 2 min, followed by denaturation at 94°C for 30 s, annealing at 55°C for 30 s, and extension at 72°C for 1 min (34 cycles).^4^ The amplified DNA was purified using a PCR purification kit (SolGent) and sequenced by Cosmo Genetech (Seoul, Korea). For bacterial species identification, the sequences were analysed using BLASTN (genome database of the National Center for Biotechnology Information, Bethesda, MD, USA).

### Evaluation of Residual Protein on Sterilised SE

The remaining 44 sterilised SEs were placed in a conical 15-ml tube (SPL; Seoul, South Korea) containing 25 ml of Phloxine B protein staining solution (Phloxine B fluorescent dye Sigma-Aldrich; St Louis, MO, USA).^27^ To confirm residual protein, a new and unused SE straight out of the package was used as a negative control and stained with Phloxine B. After staining for 10 min, the SEs were washed twice with sterile distilled water and air dried.

**Fig 1:**
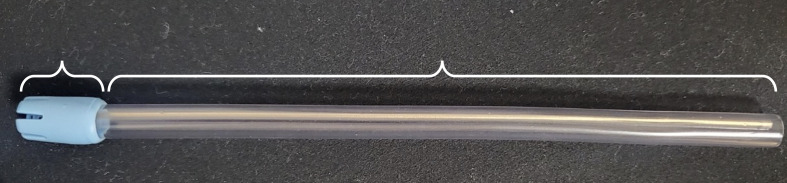
Parts of SE. T: tip; B: body (Ø6 mm x 13 mm).

Stained SEs were photographed using a digital camera and light microscope (Eclipse Ei, Nikon; Tokyo, Japan). The outer surface of the SE tip was photographed using a digital camera at 2X magnification and the presence of staining was confirmed. To determine the presence or absence of staining on the tip, the surfaces of the side, top, and surface connected to the body were evaluated; if staining was present on any of these surfaces, the final stained sample was evaluated. The clear-tube bodies of SEs (Ø6 mm x 13 mm) were photographed with a digital camera at 2X magnification on the outside, and the inside was photographed at 40X magnification using a light microscope (Nikon) to confirm the presence of staining. The area of the stained surface was measured using ImageJ software (National Institutes of Health; Bethesda, MD, USA) using the images of the stained body taken under a light microscope (Fig 1). The surface area was expressed as a percentage (%) of the total area.^18^

## RESULTS

### Residual Bacteria on Sterilised SE

**Fig 2:**
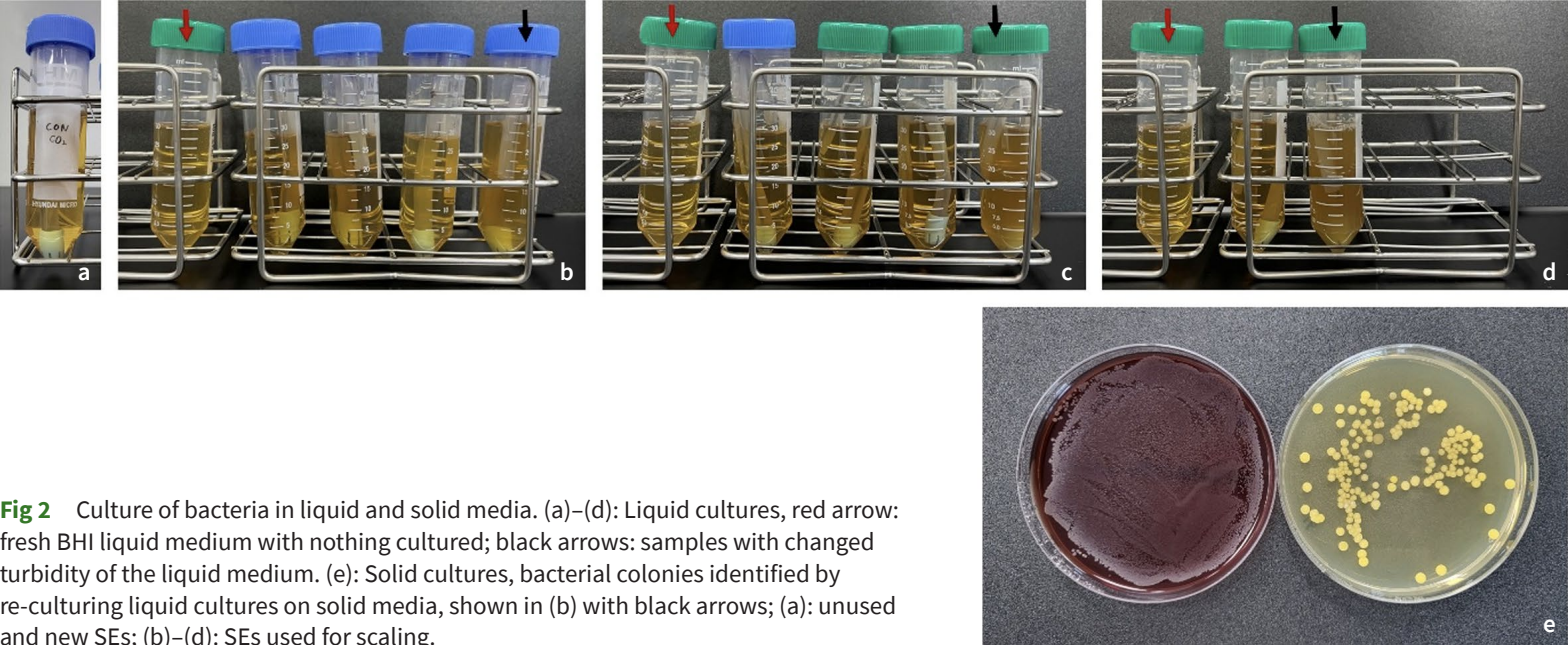
Culture of bacteria in liquid and solid media. (a)–(d): Liquid cultures, red arrow: fresh BHI liquid medium with nothing cultured; black arrows: samples with changed turbidity of the liquid medium. (e): Solid cultures, bacterial colonies identified by re-culturing liquid cultures on solid media, shown in (b) with black arrows; (a): unused and new SEs; (b)–(d): SEs used for scaling.

When the 61 SEs used in this study were incubated in BHI liquid media, three (4.92%) samples showed visible turbidity changes (Fig 2). When liquid cultures were re-cultured on solid media, only one (1.64%) sample produced bacterial colonies (Fig 2). There was no obvious visible turbidity change in the SE of the new, unused negative control.

Six bacterial colonies cultured on BHIA and BA were isolated by morphology, and species analysis revealed that all six colonies were *Staphylococcus warneri*.

### Residual Proteins on Sterilised SE

**Fig 3:**
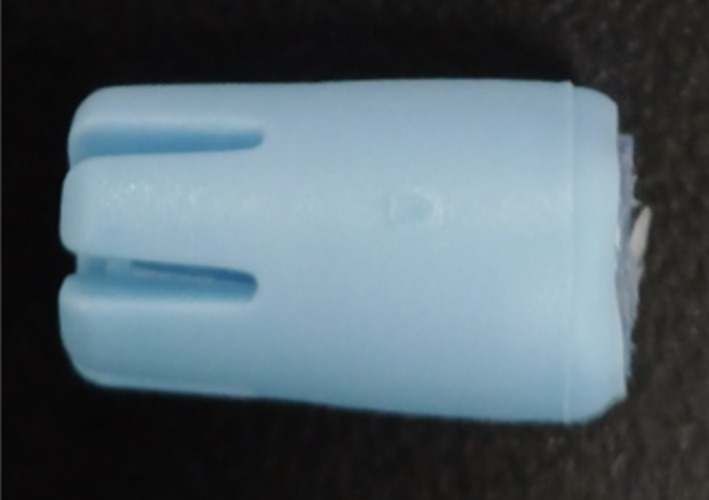

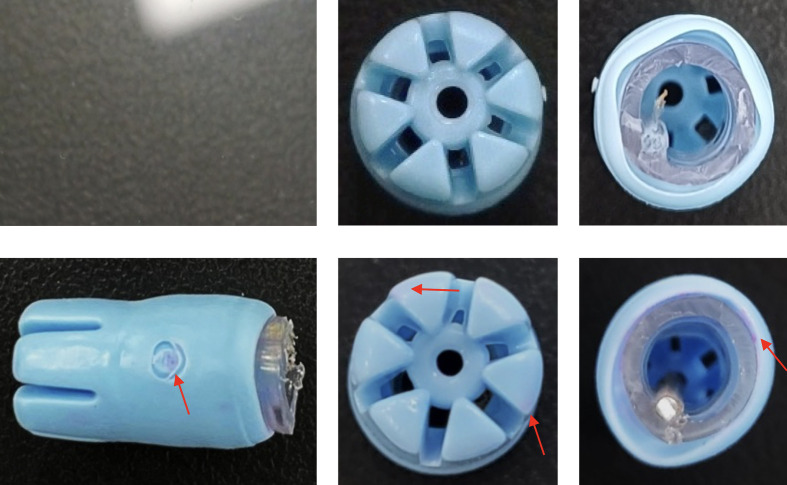
Residual protein stained at the tip site of a sterilized SE. (a)–(c): stained, unused, and new SEs; (d)–(f): SEs stained and used for scaling, red arrows: regions stained with residual protein.

**Fig 4:**
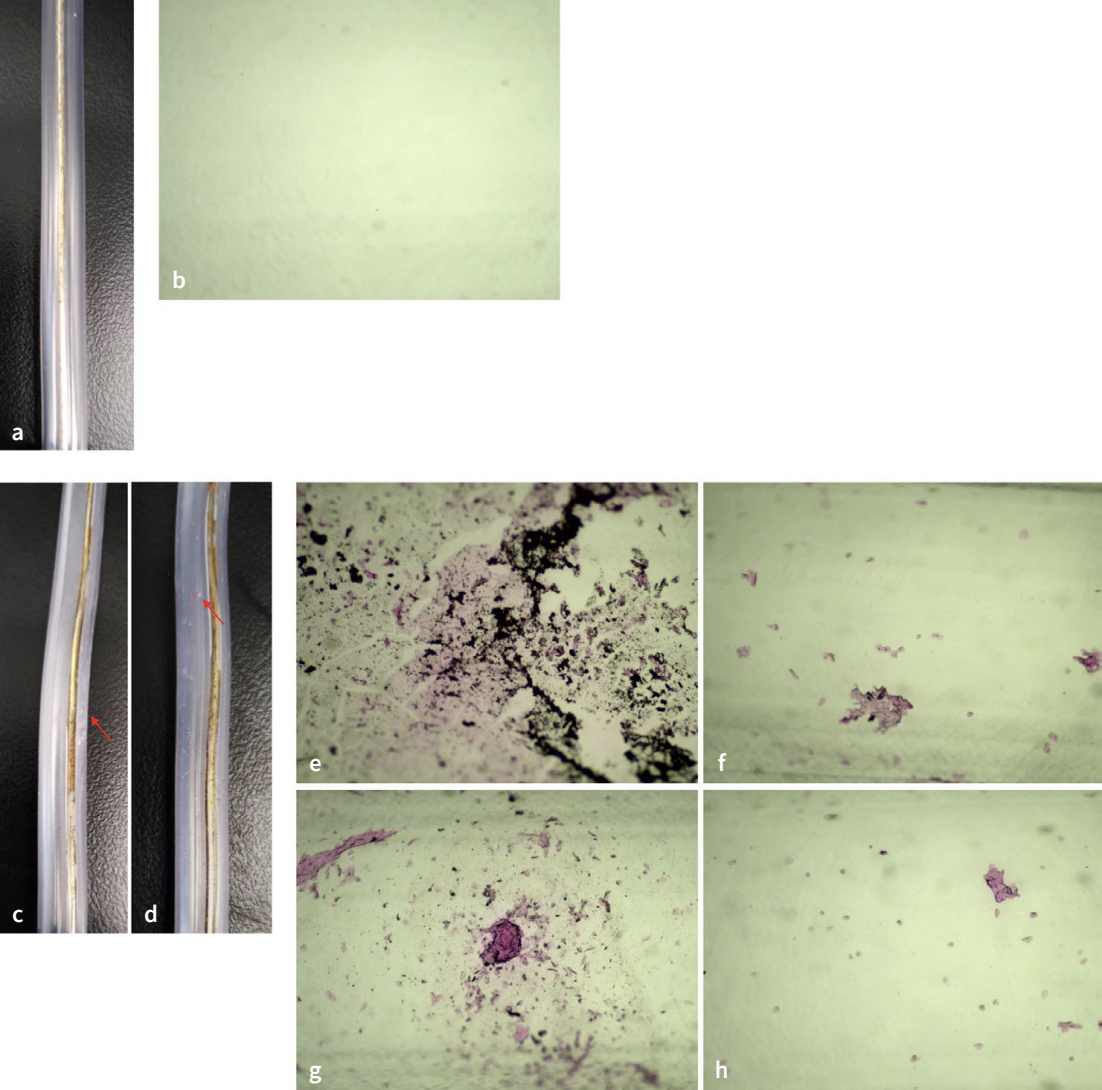
Residual protein stained in the body of the SE. (a)–(b): stained, unused, and new SEs; (c)–(h): SEs stained and used for scaling; (a), (c)–(d): A photograph of the outside of the body with a digital camera at 2X magnification; (b), (e)–(h): Photograph of the inside of the body under a light microscope at 40X magnification, red arrows: areas of residual protein stained on the inner surface of the body.

Of the 44 SEs used in the test, when observing residual protein by site, residual protein was detected in 36 (81.8%) of the samples at the tip site. Residual proteins were observed in all (100%) samples. Photographs of representative samples are shown in Figs 3 and 4. The average area of the stained residual protein in the body of each SE sample was 1.78% (standard deviation 3.1%). In contrast, new, unused SE from the negative control showed no staining at any of the sites.

## DISCUSSION

By determining the presence of bacteria and proteins, this study revealed the problems that can arise when SEs are reused after sterilisation, despite being recommended for single use by standard infection control manuals. Residual bacteria were identified in only one sample, whereas residual proteins were identified in all samples. Similarly, a previous study confirming the safety of sterilised and reused HAs found no residual microorganisms in any of the 55 HA samples used.^25^ However, the presence of organic compounds was confirmed by measuring the total organic carbon (TOC), and concerns regarding the transmission of prions and proteinaceous infectious particles were raised, emphasising the need to stop HA reuse.^25^ In a study by Wadhwani et al of 185 HAs used to determine stability after sterilisation, 66 (30.27%) HA samples were contaminated with microorganisms.^27^ The microorganisms detected were periodontal pathogenic bacteria, such as *Aggregatibacter actinomycetemcomitans* and *Prevotella intermedia,* and oral commensal bacteria, such as staphylococci.^27^ In our study, bacteria were detected in one (1.64%) sample, all of which were identified as *Staphylococcus warneri* by bacterial identification. As the experiment was conducted in a sterile environment (using a disinfected bench and sterile towels), with no skin exposure of the researcher (who wore a mask, lab gown, and disinfected gloves), and using sterile equipment, it is presumed that there was no bacterial contamination from the researcher. As Wadhwani et al^27^ also found a high number (5 out of 15) of staphylococci, it seems quite possible that staphylococci remain on dental instruments. Besides, *S. warneri* is a common commensal bacterium in human and animal skin flora, with a high prevalence in the head and nasal cavity and colony colors varying from orange to yellow to gray-white.^16^ It has also been reported to be found in the oral cavity and dental plaque, although at a lower frequency. Therefore, it is possible that the *S. warneri* identified in this study could have originated from the head, nasal cavity, or oral cavity, which may have come into contact with the SE when it was used.^5,22^ This indicates that sterilisation methods cannot completely eliminate residual microorganisms on SEs and show the potential for cross-infection owing to the reuse of SEs.

Phloxine B was used to determine the presence of residual organic compounds. Phloxine B is a ﬂuorescein derivative stain used to detect proteins and peptides, and is FDA approved for use as a drug and food coloring, as well as for bacterial staining and blood staining in forensic medicine.^24,27^ In a previous study, when Phloxine B was used to identify residual proteins on the surface of sterilised HAs, proteins were observed in 99 (99%) of 100 HAs.^27^ The detection of organic compounds, including proteins, on the surface of sterilised HA indicates its potential for prion transmission through the reuse of sterilised HA.^25,27^ Prions are proteinaceous infectious particles that can cause neurodegenerative diseases in humans, including Kuru, Creutzfeldt-Jakob disease, and variant Creutzfeldt-Jakob disease, are known to be highly resistant to proteolytic enzymes, and can survive temperatures of 200°C for 1–2 h.^12,25,27^ Even when the prions were treated with the typical sterilisation process performed in dentistry (134°C, 18 or 4 min), it was found that they were not always completely removed.^20^ This demonstrates the limitations of sterilisation for completely removing prions from the surface of dental instruments, and indicates the need for other treatment methods in addition to sterilisation to remove residual proteins and microorganisms when reprocessing dental instruments. Prions can be completely removed by some disinfectants. A previous study comparing the area of residual protein using Phloxine B after the HA had been treated with two disinfection methods, found less residual protein after disinfectant plus sonication than after water plus sonication, suggesting that the use of disinfectants can be effective in removing prions.^18,20^ However, in the same study, the screwdriver engagement sites in on the HA retained many proteins, even after a combination of disinfectants and sonication.^18^ These results demonstrate that even effective disinfectants capable of removing prions cannot absolutely remove proteins from the surface of concave sites, such as screwdriver engagement sites in HAs.

Similar to the HA’s external characteristics, the SE’s body is composed of a long tube with a narrow inner diameter and a small hollow at the tip, making it difficult to completely remove prions using the disinfection and sterilisation methods performed in dentistry. In particular, the tip is the part of the SE that comes into direct contact with the patient’s oral cavity, and the presence of residual organics on the tip exterior, as shown in this study, indicates a high risk of cross-infection when reused. In addition, Watson and Whitehouse confirmed the reflux of fluid when the SE tip was closed and sealed with the lips, creating negative pressure.^28^ This indicates the possibility that proteins residing inside the body are transferred back into the oral cavity by creating negative pressure around the tip. Therefore, it is necessary to stop SE reuse.

While the unique shapes of instruments and materials used in dentistry can affect their reprocessing, the Dental Infection Control Standards Policy Manual (Ministry of Health and Welfare, Seojong, Korea) suggests reprocessing methods based on whether the procedure involves mucosal contact and tissue penetration.^21^ For surfaces such as HA and SE where it is difficult to completely remove organics, enhanced reprocessing methods are needed, as are infection control methods that consider the shapes of instruments and materials. Some dental offices in Korea were found to reuse disposable dental materials such as prophylaxis cups, prophylaxis brushes, and irrigation needles.^23^ Analogous to the object of research in this study, it is necessary to investigate the risks of reusing these other single-use materials in the future. Because economic reasons are mentioned as one of the reasons for not practicing infection control, the economic benefits of reusing these single-use materials need to be re-assessed.^3,9,15^ Although the cost per unit of these disposable materials is low, the reuse of these materials might be perceived as a significant cost saving, considering the average number (40 on average, with a maximum of 80 or more in Korea) of patients per day.^7,9^ However, the labor costs involved in reusing them should not be overlooked, as this would make reprocessing less economical. This provides yet another reason for ceasing to reuse single-use materials.

Interest in and practice of infection control was shown to increase when there are fewer patients per dental hygienist, and when there is a designated infection control staff member.^7,10,11^ Therefore, in order to improve infection control practices, it is recommended that a related system should be established in Korea, along with research that reveals the risks of reuse.

In this regard, institutional improvements in dental device manufacturing are required. Currently, SEs, prophylaxis cups, and prophylaxis brushes, which are intended to be disposable, are packaged in bundles rather than individually.^23^ Although no residual bacteria and proteins were detected from the new SEs used as negative controls in this study, there is a high probability of contamination before they are used on patients, as dental offices keep the packages open until the contents have been completely consumed. Therefore, restrictions on the type of packaging must be required at the time of supply, and studies should be conducted to determine the extent of contamination of open disposables to highlight the need for institutional improvements.

This study has limitations in that it only confirmed the presence of proteins or peptides as organics and did not identify the properties of the stained residual proteins to accurately determine their human origin. In addition, because the bacterial colonies were separated by morphology and only six types of colonies were identified, bacteria grown in liquid culture or those not cultured on solid media were not identified.

## CONCLUSION

This is the first study to provide experimental evidence of the problems that may occur when SEs are sterilised and reused, rather than being discarded after single use, as recommended in standard infection control manuals. This study can be considered as the scientific basis for the Standard Policy Manual for Dental Infection Control. The findings of this study should raise awareness among dental professionals regarding the importance of dental infection control practices.

## ACKNOWLEDGEMENTS 

This work was supported by a National Research Foundation of Korea (NRF) grant funded by the Korean government (Ministry of Science and ICT, MSIT) (No. RS-2022-00166484).

## References

[ref1] Barbeau J, ten Bokum L, Gauthier C, Prevost AP (1998). Cross-contamination potential of saliva ejectors used in dentistry. J Hosp Infect.

[ref2] Barreiros P, Braga J, Faria-Almeida R, Coelho C, Teughels W, Souza JCM (2021). Remnant oral biofilm and microorganisms after autoclaving sterilization of retrieved healing abutments. J Periodontal Res.

[ref3] Cha SR, Kim KJ (2013). Protocol for disinfection and sterilization in dental clinic. J Korean Dent Assoc.

[ref4] de Lillo A, Ashley FP, Palmer RM, Munson MA, Kyriacou L, Weightman AJ (2006). Novel subgingival bacterial phylotypes detected using multiple universal polymerase chain reaction primer sets. Oral Microbiol Immunol.

[ref5] Fumio Nagahama OT, Uchibori S, Fukumoto M (2013). Frequency of staphylococci in oral cavity of healthy medical workers. Int J Oral-Med Sci.

[ref6] Gurevich I, Dubin R, Cunha BA (1996). Dental instrument and device sterilization and disinfection practices. J Hosp Infect.

[ref7] Hwang SH (2017). Related factors of infection control practice by dental hygienists in some areas. AJMAHS.

[ref8] Jang KA, Park JH (2016). Factors influencing infection control awareness and implementation levels among dental hygienists. J Dent Hyg Sci.

[ref9] Jeon JS, Choi SM, Lee YH (2018). A Study of differences in the infection control cognition between practice of dental hygienists. AJMAHS.

[ref10] Jeong HJ, Lee JH (2015). Impact factor of cognition and practice of infection control in the dental hygienists. J Korean Soc Dent Hyg.

[ref11] Joo SY, Shin HH, Park SS (2022). The related factors of adherence to infection prevention and control practices in dental clinics during the Coronavirus Disease 2019 Epidemic. J Health Info Stat.

[ref12] Jung MJ, Pistolesi D, Pana A (2003). Prions, prion diseases and decontamination. Ig Sanita Pubbl.

[ref13] Kang JK, Kim ES, Kim KM (2002). Study on the infection control and dental waste disposal in dental clinic located in Seoul city. J Dent Hyg Sci.

[ref14] Kim JH, Oh NR, Kim GU (2021). Correlation among sensitivity to infection, dental care avoidance, corona depression and infectious disease prevention practice during the COVID-19 pandemic. J Korean Health Fundamental Med Sci.

[ref15] Kim KM, Jung JY, Hwang YS (2007). A study on the state of infection control in dental clinic. J Korean Acad Dental Hygiene Education.

[ref16] Kloos WE, Schleifer KH (1975). Isolation and characterization of staphylococci from human skin II. Descriptions of four new species: Staphylococcus warneri, Staphylococcus capitis, Staphylococcus hominis, and Staphylococcus simulans. Int J Syst Bacteriol.

[ref17] Kohn WG, Collins AS, Cleveland JL, Harte JA, Eklund KJ, Malvitz DM (2003). Guidelines for infection control in dental health-care settings--2003. MMWR Recomm Rep.

[ref18] Kyaw TT, Nakata H, Takayuki M, Kuroda S, Kasugai S (2020). Evaluation of residual contamination on healing abutments after cleaning with a protein-denaturing agent and detergent. Quintessence Int.

[ref19] Lee YK, Kim SD (2010). About dentistry infection from dentistry medical institution recognition research of patient. J Korean Soc Dent Hyg.

[ref20] McDonnell G, Dehen C, Perrin A, Thomas V, Igel-Egalon A, Burke PA (2013). Cleaning, disinfection and sterilization of surface prion contamination. J Hosp Infect.

[ref22] Ohara-Nemoto Y, Haraga H, Kimura S, Nemoto TK (2008). Occurrence of Staphylococci in the oral cavities of healthy adults and nasal oral trafficking of the bacteria. J Med Microbiol.

[ref23] Park BY, Noh HJ (2020). Differences in dental hygienists’ infection control awareness and re-user rate of disposable dental care supplies. J Korean Soc Dent Hyg.

[ref26] Son JH, Jeong SY (2021). Dental infection control in clinical practice institutions experienced by dental hygiene students in the COVID-19 situation. J Korean Acad Dent Adm.

[ref27] Wadhwani C, Schonnenbaum TR, Audia F, Chung KH (2016). In-vitro study of the contamination remaining on used healing abutments after cleaning and sterilizing in dental practice. Clin Implant Dent Relat Res.

[ref28] Watson CM, Whitehouse RL (1993). Possibility of cross-contamination between dental patients by means of the saliva ejector. J Am Dent Assoc.

